# The Conference at Manchester

**Published:** 1921-07-02

**Authors:** 


### The Hospital

The Workers' Newspaper of Administrative Medicine and Institutional Life. Administration. National Insurance and Health.

No. 1830, Vol. LXX. SATURDAY, JULY 2, 1921. PRICE TWOPENCE

### THE CONFERENCE AT MANCHESTER.

The British Hospitals Association meets at Manchester on July 1 in even more interesting circumstances than those which attended the previous Conference in the same city ten years ago. In those days there was less belief in common policy and common action than there is at present, and though the discussions were undeniably useful and interesting, the results were less practical than they are expected to be to-day, when some form of co-operation is essential if the voluntary hospitals are to overcome the economic situation. The regional committees of the Association, though formed recently, should have prepared the way for the general conference; and there is no lack of subjects to discuss, since the Report of the Cave Commission has been barely digested hitherto. Apart from the position of the Approved Societies, the Conference is expected to discuss the question of co-ordinated appeals. On this matter, it will be remembered, the Report of the Cave Commission said: "We venture to suggest that appeals for donations should be of a more systematic character. Hospitals are rather prone to wait until a serious deficit has been incurred, and then to make a desperate effort to pay it off. We are convinced that more could be done by systematic canvassing and personal appeals regularly carried out. We further suggest that competing and overlapping appeals should not be made, and that no special appeal should be launched without consultation with the Voluntary Hospitals' Committee for the area, or in London with the King's Fund."

There are many difficulties to be confronted before such a scheme is generally adopted. Some hospitals think that they can do better by themselves than by participation in a joint appeal. Others feel that a joint appeal may have less character, and even perhaps less strict organisation than one which is started by and on behalf of a particular institution. In short, hitherto the suggestion has been only vaguely outlined, and this explains why the joint appeal has not advanced more quickly. In our view there is this principal difference between a separate and a common appeal. The first has the advantage of definite objects definitely associated with one institution. The second, since it cannot excite equal interest by a table of common hospital needs, must adopt a different method of enlisting public sympathy. It must clothe the needs of the participating hospitals in some visible and definite form, and interest the public in a shape as tangible as that of a single hospital. This was the idea behind the proposal of a Hospital Pageant, and no doubt explained why the plan has been welcomed, first, by many London hospital secretaries in the columns of The Hospital, and, later, by the three Funds. Here was a common object to work for, an object that co-operation alone could make successful. It was, in short, a form into which the desire for common action could express itself. Its advantages would be that not only would it interest the public, but teach it much about the hospitals, and thus educate the man in the street. We emphasise the suggestion again here, because we do not think that the Manchester Conference of the British Hospitals Association will come to a fruitful conclusion on the question of co-ordinated appeals, unless it is recognised that these must not be replicas of the appeal of one hospital only on a larger scale, but a new form of visible joint effort. The idea of the Hospital Pageant is the type.

There is another point which we hope will be duly appreciated. A study of the paragraph from the Report of the Cave Commission which we have already quoted shows the recommendation to be against the "appeal" in emergency as ordinarily understood, and in favour of the more systematic collection of income. Let this not be overlooked. Until 1914 the periodic appeal was, as this Report remarks, a customary source of income; and in London the chief attempt to regularise it was in the quinquennial form adopted at the London Hospital. The plan was, in fact, to make good the deficits of five years by a great quinquennial effort. It must now be recognised, we think, that such methods are growing out of date, though as long as the voluntary system endures, they will periodically be resorted to. What the Cave Commission, and what everyone else, is coming now to mean by a co-ordinated appeal is not a common and emergency effort, but a collective and systematic policy for raising income every year. For example, we cannot but think that the offices and shops in London which are largely obdurate (it is alleged) to weekly contributions, might entertain the idea more favourably if the various heads were approached by a body representing all the hospitals in London. We understand that in Manchester itself certain employers discourage the Hospital Saturday Fund, and will not allow collections. Co-operative action by all the hospitals in Manchester, together with the Saturday Fund, might persuade these employers to reconsider their attitude; a conference, at all events, would bring the reasons for it to light.

